# Routine breast milk monitoring using automated molecular assay system reduced postnatal CMV infection in preterm infants

**DOI:** 10.3389/fmicb.2023.1257124

**Published:** 2023-09-19

**Authors:** Junhyup Song, Sinyoung Kim, Eunmin Kwak, Younhee Park

**Affiliations:** Department of Laboratory Medicine, Severance Hospital, Yonsei University College of Medicine, Seoul, Republic of Korea

**Keywords:** breast milk, cytomegalovirus, Cobas 6800, preterm infants, vertical transmission

## Abstract

Human cytomegalovirus (CMV) transmitted through breast milk poses fatal risks to preterm infants. However, current molecular assay systems often do not accommodate breast milk samples. In this study, we evaluated the analytical and clinical performance of the measurement procedure of CMV load in breast milk utilizing the Cobas CMV test on the Cobas 6,800 system. This was enabled by incorporating a simple independent sample preparation procedure before the application of samples on the automated assay system. Clinical data from electronic medical records were retrospectively analyzed. Breast milk samples from mothers of preterm infants born before 33 weeks of gestation were screened for CMV using the automated assay system. CMV positivity rates in breast milk and neonatal samples and the CMV transmission rate were calculated. Furthermore, to validate the analytical accuracy of the overall measurement procedure with newly obtained residual breast milk samples, the linearity of the measurement procedure was assessed, and a simplified sample preparation method was validated against a conventional method. The CMV positivity rates in maternal breast milk and neonatal samples were 57.8 and 5.2%, respectively. The CMV transmission rate through breast milk was 7.7%. No significant differences in gestational age or birth weight were found between the CMV-negative and CMV-positive neonates. The linearity of the procedure was observed within a range of 1.87–4.73 log IU/mL. The simplified sample preparation method had an equivalent or even improved CMV detection sensitivity than the conventional method. Incorporating a simple independent sample preparation procedure effectively resolved any potential issues regarding the application of breast milk on the automated assay system. Our approach contributed to reduced vertical transmission of CMV by providing a convenient and reliable method for the monitoring of breast milk CMV positivity for clinicians.

## 1. Introduction

Similar to other human herpesviruses, human cytomegalovirus (CMV) can persist within human cells following acute infection in the host (Landolfo et al., 2003). Although the worldwide prevalence of CMV seropositivity is estimated to be 83%, severe symptoms resulting from primary CMV infection are rarely reported in immunocompetent individuals (Ho, 1990; Wreghitt et al., 2003). However, this does not hold for immunocompromised individuals, including patients with human immunodeficiency virus, post-transplant patients, as well as fetuses and neonates. CMV infection in fetuses can lead to devastating consequences, such as microcephaly, developmental delay, hearing impairment, and visual deficits (Malm and Engman, 2007).

Although early postnatal CMV infection, which is transmitted through breast milk, rarely leads to long-term neurodevelopmental defects, it can cause death in preterm or low-birth-weight infants through CMV-related sepsis-like syndrome (CMV-SLS) (Lanzieri et al., 2013). Breast milk was first recognized as a potential route of CMV transmission in the 1970s (Ballard et al., 1979; Stagno et al., 1980). Since then, the presence of CMV in breast milk and its association with infantile infection has been investigated using viral culture and polymerase chain reaction techniques (Dworsky et al., 1983; Hotsubo et al., 1994). Viral reactivation in breast milk has previously been reported in over 90% of seropositive mothers (Hamprecht et al., 2001; Meier et al., 2005). Moreover, breastfeeding from seropositive mothers has resulted in CMV infection in 10–40% of preterm infants and CMV-SLS in 2–5% of cases (Hamprecht et al., 2001; Mussi-Pinhata et al., 2004; Capretti et al., 2009). Although current guidelines generally overweigh the benefits of regular breastfeeding over the risk of CMV transmission in preterm infants, the risk of CMV-SLS cannot be ignored (Eidelman et al., 2012).

The ever-increasing need for routine laboratory tests, including molecular diagnostic tests for infectious agents, has resulted in the widespread adoption of automated assay systems in clinical laboratories. However, these high-throughput, automated systems have some drawbacks, including a reduced level of versatility. For instance, the Cobas CMV test using the Cobas 6,800 system is specifically designed for ethylenediaminetetraacetic acid plasma samples (Roche-Diagnostics-GmbH, 2023). This approach facilitates efficient and error-free batch-based high-throughput testing; however, the clinical need for diagnostic tests that utilize diverse sample types, such as cerebrospinal fluid, sputum, breast milk, or pus, has not actively been addressed (Guo and Jiang, 2019; Hernandez-Alvarado et al., 2021; Inoue et al., 2021; Lee et al., 2022b).

In this study, we report our three-year experience of detecting CMV in the breast milk of premature babies using an automated molecular assay system. We believe that CMV detection in breast milk has proven valuable for clinicians in determining the appropriate timing for initiating breast milk feeding. Consequently, this approach has contributed to a reduced CMV infection rate and lower incidence of CMV-SLS.

## 2. Materials and methods

### 2.1. Study design

In this retrospective observational study, we conducted analyses using data obtained from electronic medical records at Severance Hospital, a tertiary hospital in Korea. Data from the study participants included the sex; gestational age at birth; birthweight; CMV PCR results from serum, urine, or breast milk samples; CMV IgM and IgG titer; and day of initiation of breastfeeding.

The neonatal intensive care unit in the hospital has a policy regarding the monitoring of CMV transmission via breast milk, as described below:

- All the breast milk is frozen and thawed before feeding to the neonates. This process aims to reduce the viral load and facilitate efficient storage and timely feeding.
- Breast milk for preterm babies born before 33 weeks of gestation is subjected to a CMV PCR assay. If the result is positive, breast milk feeding is withheld until 35 weeks of gestational age (GA). Otherwise, breast milk feeding is initiated as soon as the baby is ready.
- For preterm babies born after 33 weeks of gestation, breast milk feeding is recommended without any limitations.

All preterm infants born before 33 weeks of GA who were tested for CMV infection at least once using plasma or urine samples and whose mother’s breast milk was tested for CMV detection were included in this study. A neonate was deemed CMV-positive when at least one positive result was observed from the plasma or urine sample test. As a result, we obtained a cohort of 135 pairs of neonates and mothers. Using this study population, we analyzed CMV positivity and viral load in breast milk according to the age of the neonate. Furthermore, we estimated the transmission rate from mothers with CMV-positive breast milk. Additionally, we compared the birthweight, gestational age at birth, and CMV positivity rate in maternal breast milk between CMV-positive and CMV-negative neonate groups.

Furthermore, we evaluated the linearity and analytical sensitivity of our measurement procedure, which included an independent sample preparation step before the application of the breast milk samples on the Cobas 6,800 system. The analytical performance of the assay was evaluated using several randomized samples.

### 2.2. Ethical approval

This study protocol was reviewed and approved by the institutional review board of the Severance Hospital, Seoul, Korea (IRB No. 4–2023-0414). The anonymized data from medical records were retrospectively obtained and analyzed. The analytical validation of the measurement procedure was conducted using de-identified random residual breast milk samples. Due to this design of our study, informed consent was waived, as privacy was thoroughly protected and the study involved minimal risk to patients.

### 2.3. Simplified sample preparation

The breast milk samples were pre-processed to effectively reduce lipid and cellular components and minimize additional labor. Briefly, the samples were centrifuged at 2500 g for 15 min to obtain the supernatant without the creamy layer. Subsequently, a 10-fold dilution was performed using distilled water.

To assess the viability of this simplified preparation method, it was compared to a more elaborate method described previously (Hamprecht et al., 1998). In this previously established method, breast milk samples were centrifuged at 400 g for 10 min, and the creamy top layer was discarded. Subsequently, centrifugation was performed at 400 g and 3,200 g for 10 min each. The resulting supernatant was then collected and filtered through a 0.22 μm-pore-size filter. Finally, a 10-fold dilution was performed using distilled water.

### 2.4. Cobas 6,800 CMV test

The Cobas 6,800 system is a fully integrated molecular assay system comprising two modules: nucleic acid extraction and PCR amplification and analysis. Viral nucleic acid is extracted using proteinase and lysis reagents. Thereafter, the released nucleic acid binds to magnetic glass particles. Following the washing out of cellular debris and denatured proteins, the purified nucleic acid is eluted from the glass particles using an elution buffer. The amplification of nucleic acid is based on virus-specific forward and reverse primers that target a highly conserved region of the CMV DNA polymerase UL54 gene. To quantify the viral load, the amplification of DNA-QS, a non-CMV DNA quantitation standard, is performed in parallel. The assay employs one detection probe for the CMV target sequence and another for the DNA-QS. During the PCR amplification process, the probe hybridizes to the specific target sequence, causing the fluorescent reporter dye and quencher dye to separate. By measuring and comparing the released signal from the viral target and DNA-QS, the system achieves quantification of the viral load. DNA-QS also serves as the internal standard for the assay. When the measurement value of DNA-QS deviates from the predefined allowable range, an “invalid” result is reported rather than the calculated IU/mL value for the target viral load.

Plasma was prepared from whole blood samples collected in EDTA tubes containing a separator gel. The samples were centrifuged at 2500 g for 15 min to isolate the plasma. Urine samples were collected in conical tubes and then centrifuged at 2500 g for 10 min; the supernatant was then subjected to the measurement. Breast milk samples were processed following the method outlined earlier in this section. A specimen is considered positive if the CMV DNA load exceeds 34.5 IU/mL, which is the limit of quantification (LOQ) for this assay.

### 2.5. Analytical performance of the procedure

The linearity of the Cobas CMV test, following the simplified sample preparation step, was verified following the Clinical & Laboratory Standards Institute guideline (CLSI) EP06-A. A random breast milk sample with a CMV viral load of approximately 5.41 × 10^4^ IU/mL was utilized. To include seven concentrations within the targeted linearity interval, with equal spacing in log concentration, the sample was serially diluted by a factor of 3, until 3^8^, with distilled water. The CMV viral load was then measured per sample dilution in triplicates using the Cobas 6,800 system. Subsequently, the average log-transformed CMV titers obtained from the serial dilution were examined for linearity. A maximum allowable deviation of 10% from the expected value was applied during the assessment.

The limit of detection (LOD) was verified following the CLSI EP17-A2 guidelines. For this evaluation, two distinct breast milk samples, each harboring a CMV load of 20.6 IU/mL, which was the LOD claimed by the manufacturer, were prepared. These samples were further divided into 10 replicates each. Subsequently, each replicate was measured across 3 days. Given that a total of 20 measurements were conducted, the threshold for observed proportion was set to 85% during the assessment.

The cross-reactivity of the assay was evaluated in our previous study (Roh et al., 2021).

### 2.6. Statistical analysis

The transmission rate of CMV was determined as the number of CMV-positive neonates from breast milk CMV-positive mothers over the total number of breast milk CMV-positive mothers. Continuous variables were compared using an independent t-test, while categorical variables were compared using Fisher’s exact test. All the statistical analyses and data visualization were performed using SPSS version 26.0 (IBM Corp., Armonk, NY) and GraphPad Prism version 9 (GraphPad Software, La Jolla, United States). *p* < 0.05 was considered statistically significant.

## 3. Results

### 3.1. Subject demographics

**Figure 1:**
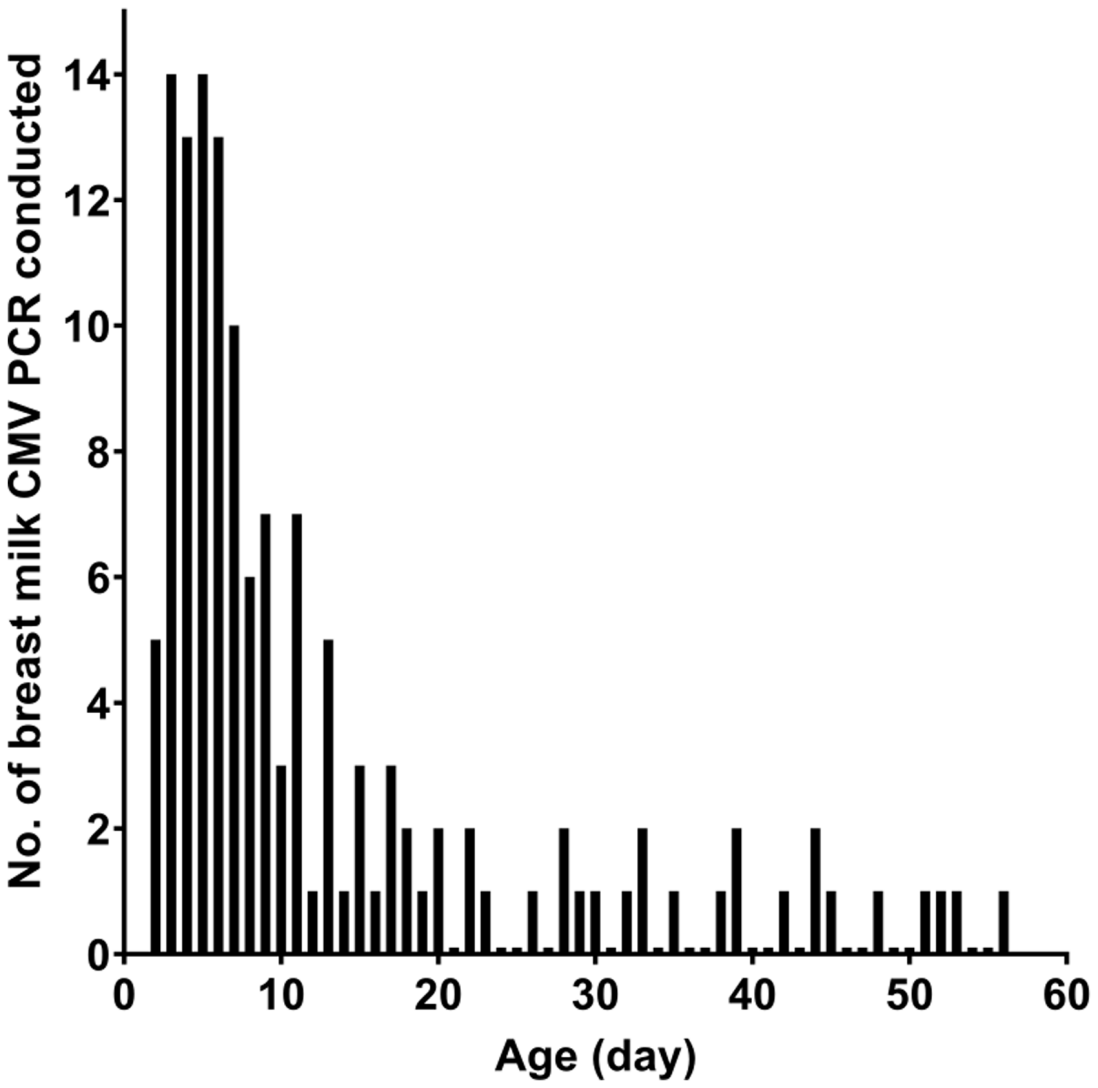
Number of conducted CMV PCR according to the age of the neonate.

**Figure 2:**
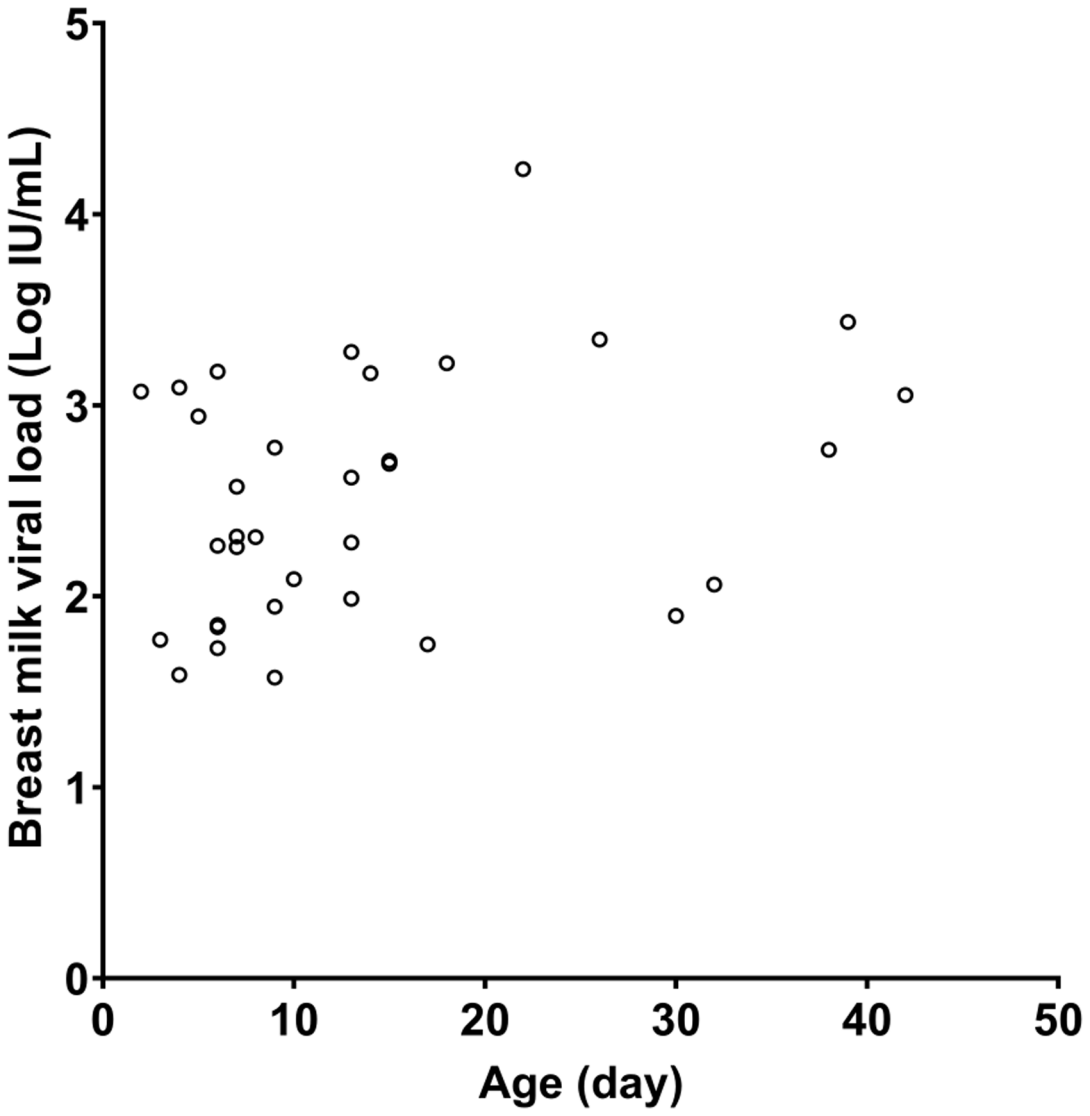
CMV viral load in maternal breast milk according to the age of the neonate. Only quantitative results from positive samples are presented.

Table 1 presents the characteristics of the study subjects. The preterm infants had an average gestational age of 28.5 weeks at birth and an average birth weight of 1,150.5 g. In most cases, CMV infection in neonates was screened using urine samples. Most of the CMV PCR assays on breast milk were conducted within the first week after birth (Figure 1). Notably, the CMV viral load in breast milk gradually increased following birth (Figure 2). It is worth mentioning that the initiation of breastfeeding differed among the neonates based on the CMV positivity of their mother’s breast milk (Supplementary Table S1).

**Table 1:** Characteristics of the study subjects.

Parameters	Study subjects (*n* = 135)
Sex, male (%)	74 (54.8)
Gestational age at birth, weeks*	28.5 (28.1–28.9)
Birthweight, g*	1,150.5 (1,080.2–1,220.8)
Breast milk positivity (%)	78/135 (57.8)
Breast milk viral load, IU/mL**	<34.5–17,200.0
Plasma positivity (%)	5/13 (38.5)
Plasma viral load, IU/mL**	<34.5–1,360.0
Urine positivity (%)	7/135 (5.2)
Urine viral load, IU/mL**	<34.5–2,670,000.0

*Means with 95% confidence intervals.

**Minimum and maximum values.

IU, international unit.

### 3.2. CMV transmission rate

Table 2 presents the vertical transmission rates of CMV observed in the present and previous studies (Omarsdottir et al., 2015; Hernandez-Alvarado et al., 2021; Lee et al., 2022a). In our study, the overall CMV positivity rate in maternal breast milk and neonatal urine samples was 57.8 and 5.2%, respectively. The transmission rate through breast milk was 7.7%.

**Table 2:** CMV transmission rate via breast milk.

	Year of publication	Number of subjects	Breast milk positivity, %	Neonate CMV positivity, %	Transmission rate, %*
Present study	–	135	57.8	5.2	7.7 (6/78)
Lee et al.	2022	147	68.0	11.6	17.0 (17/100)
Hernandez-Alvarado et al.	2021	150	43.0	4.9	13.6 (9/66)
Omarsdottir et al.	2015	140	59.5	3.6	8.1 (3/37)

*The transmission rate of CMV was determined as the number of CMV-positive neonates from breast milk CMV-positive mothers over the total number of breast milk CMV-positive mothers.

### 3.3. CMV-negative and-positive neonates

The characteristics of CMV-negative neonates (i.e., sex, gestational age at birth, birthweight, and maternal breast milk positivity) were not significantly different from those of CMV-positive neonates (Table 3). Of the 135 neonates, seven were CMV-positive, all of whom had mothers with CMV-positive breast milk, except for a single case. Meanwhile, among the CMV-negative neonates, 72 (56.3%) had mothers with CMV-positive breast milk.

**Table 3:** Association between various factors and the CMV-positivity of neonates.

	CMV-negative neonates	CMV-positive neonates	*p*-value
Number	128	7	–
Sex, male (%)	71 (55.5)	3 (42.9)	0.701
Gestational age at birth, weeks*	28.5 (28.1–28.9)	28.7 (26.7–30.7)	0.817
Birthweight, g*	1145.8 (1072.8–1218.8)	1237.1 (930.0–1544.3)	0.571
Breast milk positivity (%)	72 (56.3)	6 (85.7)	0.238

*Means with 95% confidence intervals.

### 3.4. Analytical performance of the procedure

**Figure 3:**
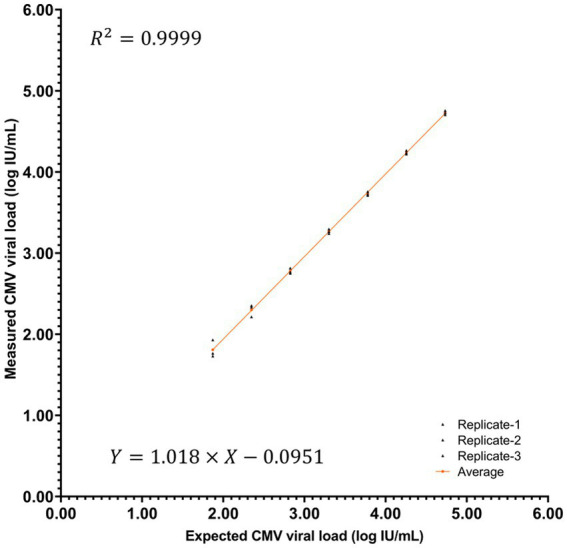
Linearity curve showing the log-transformed CMV titer of the raw or diluted breast milk samples.

As per the linearity experiment, the average log-transformed values of the selected breast milk sample concentrations serially diluted with distilled water were 4.73, 4.24, 3.73, 3.27, 2.78, 2.30, and 1.81 (Figure 3). The measured titer at a 2,178-fold (3^7^) dilution was below the LOQ. Linearity was observed within a range of 1.87–4.73 log IU/mL, with a coefficient of determination of 0.9999.

In verifying the LOD, the target DNA was detected in 17 of 20 measurements (Table 4). This result verified that the Cobas CMV test can detect CMV DNA at a concentration of 20.6 IU/mL.

**Table 4:** Verification of the limit of detection for the Cobas CMV test.

	Sample 1				Sample 2			
	Rep-1	Rep-2	Rep-3	Rep-4	Rep-1	Rep-2	Rep-3	Rep-4
Day 1	< Titer min*	< Titer min	< Titer min	TND	< Titer min	< Titer min	< Titer min	38.1 IU/mL
Day 2	TND	< Titer min	43.0 IU/mL	< Titer min	TND	< Titer min	< Titer min	< Titer min
Day 3	< Titer min	< Titer min	–	–	< Titer min	< Titer min	–	–

*The results below the limit of quantitation were reported as < Titer min.

Rep, replicate; TND, target not detected.

We previously verified that the Cobas CMV test exhibits no cross-reactivity with clinically significant viruses, including Epstein–Barr virus, herpes simplex virus, BK virus, adenovirus, hepatitis B and C viruses, human immunodeficiency virus, human papillomavirus, enterovirus, rhinovirus, influenza A and B viruses, respiratory syncytial virus, bocavirus, and varicella-zoster virus (Roh et al., 2021).

### 3.5. Comparison of results based on the sample preparation processes

Compared to the previous sample preparation method of Hamprecht et al. (1998), our simplified sample preparation method had an equivalent or even improved CMV detection sensitivity using breast milk samples (Supplementary Table S2). In addition to the negative-to-positive conversion of one sample, we observed that five samples with CMV titers higher than the LOD exhibited a substantially increased CMV load, approximately tenfold in magnitude.

## 4. Discussion

Due to the frequent presence of viremia or viruria in symptomatic CMV infections, plasma and urine have often been the primary specimens used for CMV detection. However, recent studies have highlighted the use of non-typical specimens, including the saliva of neonates and bronchial lavages and tracheal aspirates of patients with CMV pneumonia (Dunn et al., 2023; Lima et al., 2023). In this study, we assessed the effectiveness of the Cobas 6,800 system for the detection of CMV using breast milk. This fully integrated system offers enhanced testing capacity, which we believe will result in the more active monitoring of CMV positivity in maternal breast milk and ultimately contribute to a significant reduction in the incidence of CMV-SLS cases.

There are various methods for inactivating CMV in breast milk, and the most widely accepted method is pasteurization. Pasteurization typically involves heating the breast milk at 62–72°C (Björkstén et al., 1980; Goldblum et al., 1984). Long-term pasteurization ensures the complete eradication of CMV; however, it can also significantly reduce the nutritional and immunologically beneficial components of breast milk (Liebhaber et al., 1977). In contrast, short-term pasteurization aims to preserve the quantity and functionality of these beneficial components while effectively inactivating CMV (Goldblum et al., 1984). Another commonly used method is freeze-thawing of breast milk before feeding it to preterm infants. While the effectiveness of this method in reducing CMV is not fully guaranteed and conflicting opinions surround its efficacy, freeze-thawing minimally affects the nutritional and immunological properties of breast milk (Friis and Andersen, 1982; Hamprecht et al., 2004; Maschmann et al., 2006). In general, frozen–thawed breast milk has a lower rate of CMV transmission than untreated breast milk (Hu et al., 2021). It is noteworthy that the subjects in our study showed a transmission rate of 7.7%, which is equivalent to the lowest rates observed in previous studies where subjects were given frozen–thawed maternal breast milk. This result suggests the effectiveness of the test-and-initiate strategy in determining the appropriate timing for initiating breastfeeding to minimize CMV transmission.

The 2012 policy statement of the American Academy of Pediatrics stated that the value of routinely feeding human milk from seropositive mothers to preterm infants outweighs the risks of clinical disease, especially because no long-term neurodevelopmental abnormalities have been reported (Eidelman et al., 2012). In line with this, the revised statement in 2022 emphasized that maternal breast milk for very low birth weight infants should be considered a medical therapy, with higher doses associated with maximal health benefits (Meek et al., 2022). Thus, we propose that our test-and-initiate strategy can effectively minimize the risk of CMV-SLS while simultaneously maximizing the advantages of breast milk. For instance, breastfeeding can be withheld only when maternal breast milk has a very high CMV titer, especially in cases of preterm infants with specific risk factors, such as gestational age under 30 weeks or birth weight under 1,000 g. Furthermore, to provide safer monitoring, the maternal breast milk could be checked for CMV reactivation before every next breastfeeding occasion to identify any negative-to-positive conversion after the first feeding.

Our simplified sample preparation method demonstrated sufficient sensitivity for detecting CMV in breast milk. Previous studies have indicated that milk whey from breast milk is most suitable for CMV detection and plays a crucial role in the vertical transmission of CMV (Asanuma et al., 1996; Hamprecht et al., 1998). However, the lipid component of breast milk, along with lactoferrin, can inhibit PCR amplification of the target DNA (Hamprecht et al., 1998; Al-Soud and Rådström, 2001). Furthermore, the separation of breast milk components is challenging due to the sticky and thick nature of the lipid component. Therefore, obtaining the milk whey portion with minimal contamination was performed with the utmost care. Our simplified preparation method resulted in a significantly higher viral load in most of the positive samples, which was 5–10 times higher than that obtained using the previous sample preparation method of Hamprecht et al. (1998). One possible explanation for the improved sensitivity of our preparation method could be the omission of filtering through a 0.22 μm pore size filter. The inclusion of a small amount of breast milk cells may be tolerated by the automated DNA extraction module and provide a substantial amount of additional target CMV DNA.

Our study has several limitations. Firstly, the retrospectively obtained clinical data of the study subjects could not sufficiently represent CMV-positive preterm infants. The associations between CMV positivity and several factors such as gestational age at birth, birth weight, and CMV viral load in breast milk have been previously and consistently observed (Maschmann et al., 2001; Lanzieri et al., 2013). These associations might not be evident in our study due to the small number of CMV-positive samples included. Secondly, there were some missing CMV titer data for breast milk samples, thus further restricting the size of analyzable data and preventing a comparison between CMV viral loads in maternal breast milk of CMV-positive and-negative infants. Thirdly, since maternal CMV status was not universally investigated, other routes of vertical CMV transmission could not be excluded. However, CMV transmission through breast milk has been reported as the predominant mode of vertical transmission in regions with high CMV seroprevalence, such as South Korea (Chung et al., 2023).

Further research with a larger sample size is expected to demonstrate that quantitative CMV viral loads from an automated assay system can be utilized as a predictor of CMV transmission. Additionally, the test-and-initiate breastfeeding strategy can be further refined. Prospective research with the incidence of CMV infection or CMV-SLS as an endpoint could be helpful to determine the most effective criteria for preterm infants for conducting maternal breast milk CMV tests.

In conclusion, we have described our test-and-initiate breastfeeding strategy for CMV-seropositive mothers and preterm infants. This approach employed an automated molecular assay system for the routine CMV screening of breast milk. A simple, independent breast milk preparation process was used in the automated molecular assay system. We hope that further research with a larger sample size will corroborate these findings and establish the significance of the CMV viral load obtained through the automated assay system as a predictive factor for the vertical transmission of CMV in preterm infants.

## Data availability statement

The raw data supporting the conclusions of this article will be made available by the authors, without undue reservation.

## Ethics statement

The studies involving humans were approved by the institutional review board of the Severance Hospital, Seoul, Korea. The studies were conducted in accordance with the local legislation and institutional requirements. The ethics committee/institutional review board waived the requirement of written informed consent for participation from the participants or the participants’ legal guardians/next of kin because Due to the design of our study, informed consent was waived, as privacy was thoroughly protected and the study involved minimal risk to patients.

## Author contributions

JS: Writing – original draft. SK: Supervision, Writing – review & editing. EK: Data curation, Investigation, Writing – review & editing. YP: Conceptualization, Formal analysis, Supervision, Writing – review & editing.

## Funding

The author(s) declare that no financial support was received for the research, authorship, and/or publication of this article.

## Conflict of interest

The authors declare that the research was conducted in the absence of any commercial or financial relationships that could be construed as a potential conflict of interest.

## Publisher’s note

All claims expressed in this article are solely those of the authors and do not necessarily represent those of their affiliated organizations, or those of the publisher, the editors and the reviewers. Any product that may be evaluated in this article, or claim that may be made by its manufacturer, is not guaranteed or endorsed by the publisher.
