# Supplementary material for: Routine breast milk monitoring using automated molecular assay system reduced postnatal CMV infection in preterm infants
Source: Front Microbiol. 2023 Sep 19;14:1257124. doi: 10.3389/fmicb.2023.1257124 (PMC10546183; doi:10.3389/fmicb.2023.1257124)
Supplement: Supplementary file 1 [file Table_1.DOCX]

Supplementary Material

# Supplementary Tables

**Supplementary Table 1.** Initiation of breastfeeding for neonates according to the CMV status of maternal breast milk

|  | CMV-negative breast milk | CMV-positive breast milk | P-value |
| --- | --- | --- | --- |
| Age, days | 15.6 (11.3–19.8) | 34.1 (27.8–40.4) | <0.001 |
| Gestational age, weeks | 31.1 (30.4–31.9) | 33.1 (32.2–34.0) | 0.001 |
| ICU-day, days | 14.0 (10.3–17.7) | 29.6 (24.5–34.7) | <0.001 |

Data are presented as means with 95 % confidence intervals.

**Supplementary Table 2.** Comparison of results between clinical samples prepared using the reference method and our simplified method

| Sample ID | Reference method | Simplified method |
| --- | --- | --- |
| 1 | Target Not Detected | <35 |
| 2 | Target Not Detected | Target Not Detected |
| 3 | Target Not Detected | Target Not Detected |
| 4 | 5.54 × 10 | 5.94 × 10^2^ |
| 5 | Target Not Detected | Target Not Detected |
| 6 | Target Not Detected | Target Not Detected |
| 7 | Target Not Detected | Target Not Detected |
| 8 | 3.09 × 10^2^ | 2.58 × 10^3^ |
| 9 | 2.17 × 10^3^ | 3.58 × 10^4^ |
| 10 | <35 | 1.29 × 10^2^ |
| 11 | <35 | 1.21 × 10^2^ |
| 12 | Target Not Detected | Target Not Detected |
| 13 | Target Not Detected | Target Not Detected |

Data are presented as IU/mL.
